# Vasculogenesis of decidua side population cells of first-trimester pregnancy

**DOI:** 10.1186/scrt200

**Published:** 2013-05-07

**Authors:** Qiushi Wang, Licong Shen, Wei Huang, Yong Song, Li Xiao, Wenming Xu, Ying Liu

**Affiliations:** 1 Department of Obstetrics and Gynecology, West China Second University Hospital of Sichuan University, Renminnalu 3 Duan 20hao, Chengdu, Sichuan 610041, China; 2 The Chinese University of Hong Kong Joint Laboratory for Reproductive Medicine, Shatin Hong Kong; 3 Key Laboratory of Obstetric, Gynecologic and Pediatric Diseases and Birth Defects of Ministry of Education, West China Second University Hospital of Sichuan University, Renminnanlu 3Duan 19hao, Chengdu, Scihuan 610041, China

**Keywords:** Human decidua, Side population cells, Differentiation potential, Neovascularization

## Abstract

**Introduction:**

Sufficient uterine blood supply is essential for the fetus to develop normally in the uterus. Several mechanisms are involved in the process of vessel development in deciduas and villus. We focus on whether first-trimester decidua side population (SP) cells contain cells capable of differentiating into endothelial cells.

**Methods:**

Eight decidua samples were collected from healthy women, 22- to 30-years old, undergoing elective terminations of early pregnancy (six to eight gestational weeks). The cell suspensions from human deciduas were stained by Hoechst 33342 and sorted by flow cytometry, further cultured under differentiation conditions and analyzed for specific markers. These cells were implanted into ischemic limbs of nude mice to test the capacity of angiogenesis *in vivo* by DiI tracers and immunohistochemistry.

**Results:**

Decidua CD31^-^CD146^-^ SP cells of first-trimester human pregnancy can differentiate into endothelial cells, express the corresponding specific markers of endothelial cells, such as CD31 and CD146, and form tube-like structures on Matrigel and part of newly formed vessels in the ischemic limbs of nude mice. Vascular endothelial growth factor was more effective in promoting proliferation of CD31^-^CD146^-^SP cells compared with other growth factors, and estrogen and progesterone at a final concentration of 10 μmol/L and 30 μmol/L, respectively, promoted the migration of CD31^-^CD146^-^SP cells in a dose-dependent manner.

**Conclusions:**

CD31^-^CD146^-^ SP cells may be involved in the formation of new vessels in the maternal aspect of the placenta in the first trimester.

## Introduction

Sufficient nourishment is essential for a fetus to develop normally in the uterus. Abnormal uterine blood supply is associated with higher miscarriage, preterm delivery, preeclampsia and intrauterine growth restriction [1]–[3]. To meet the rising needs of the fetus, both vasodilation and development of new vessels occur during pregnancy. Evidence from several studies confirms that the vascular density of decidua tissues increases quickly in the first trimester and continues to increase slowly throughout gestation [4]–[6]. Many research studies have described the development of new blood vessels in the early placenta, especially the fetal aspects, in detail including the formation mechanism, the molecular profile changes of the endothelial complexes and their molecular regulation, and the respective growth factors [7]–[9]. The maternal aspect of the placenta was usually reputed to be the remodeling of the maternal uterine vessels [10]. Actually, the endometrium is rich in stem cell-like cells [11]–[13]. Since side population (SP) cells were found by Goodell and used to isolate these stem cell-like cells [14], several researchers have focused on the SP cells in the human endometrium [15,16], and they found that these cells could differentiate into adipose cells, bone cells and endothelial cells. However, research on decidua cells in early pregnancy has been rare. Our previous work identified stem/progenitor cells in the deciduas of human first-trimester fetuses by using fluorescent Hoechst dye 33342 to isolate SP cells. These SP cells have been proven to form clones and have differentiated into decidua mesenchymal cells [17,18]. Recently, we found that the subfraction of CD31^-^CD146^-^ SP cells revealed distinct properties, and differentiated into endothelial cells and can be promoted by vascular endothelial growth factor (VEGF), estrogen and progesterone *in vitro*. In addition, the CD31^-^CD146^-^SP cell subfraction caused functional revascularization in hind limb ischemia *in vivo*. Therefore, SP cells in decidua may play a role in the process of new blood vessel development in the maternal placenta. In this study, we investigated the different factors required for the induction of CD31^-^CD146^-^ SP cells to endothelial cells *in vitro* and confirmed the findings in further animal experiments.

## Methods

### Study population

This study was performed in accordance with the Declaration of Helsinki and approved by the Medical Research Review Board of West China Second University Hospital of Sichuan University (2009023). Eight healthy women at six to eight weeks gestation who sought a surgical termination of pregnancy for personal reasons were enrolled in the study. Gestational age was calculated from the last menstrual period and confirmed by ultrasound measurements of the gestational sac and fetal bud (the fetal bud was seen in three cases). Clinical details were recorded for each woman; they were 22– to 30-years old and had regular menstrual periods and a normal pregnancy without any pregnancy-related disorders or any medicine usage within the prior three months. Each woman gave signed informed consent.

The nude mice used in this study were five- to six-week-old, healthy, weighed 16 to 18 g, and were housed and fed in a specific-pathogen free (SPF) environment. The study was approved by the ethical committee of West China Second University Hospital of Sichuan University.

### Flow cytometry

The primary decidua cells from human first-trimester fetuses (n = 8) were separated, cultured for 24 to 48 hours and then digested and labeled with Hoechst 33342 (Invitrogen, Paisley, UK) as previously described [17,18]. Then the cells were incubated with mouse anti-human CD31 (fluorescein isothiocyanate (FITC), BD Biosciences San Jose, CA, USA) and mouse anti-human CD146 (phycoerythrin (PE), BD Biosciences) for 20 minutes at 4°C. Analysis/sorting of cells was performed using a flow cytometer BD aria2 special order containing 355 UV (BD Biosciences).

### Cell cultures

We adopted suitable culture medium conditions to maintain the sorted CD31^-^CD146^-^ SP cells, using EBM2 (Lonza Walkersville, MD, USA), including growth factors such as insulin-like growth factor (IGF) -1 and epidermal growth factor (EGF) [19]. The medium used for inducing SP cells into endothelial cells was basic fibroblast growth factor (bFGF) and vascular endothelial growth factor (VEGF) –A. The cell fraction was plated onto collagen type I-coated dishes (BD Biosciences) in EBM2 supplemented with suitable growth factors. The medium was changed every four to five days. Once cells reached 50% to 60% confluence, they were detached by incubation with 0.25% trypsin containing 0.02% ethylenediaminetetraacetic acid (EDTA) at 37°C for 5 minutes and subcultured at a 1:3 dilution under the same conditions for more than 20 passages.

### Proliferation, chemotaxis and migration assay

To measure proliferation of CD31^-^CD146^-^ SP cells compared with non-SP cells, at the third passage (at 10^3^ cells per 96-well plate) these cells were cultured in EBM2 supplemented with 0.2% fetal bovine serum (FBS, GIBCO BRL, Gaithersburg, MD, USA) and bFGF (50 ng/ml; R&D Systems, Minneapolis, MN, USA), VEGF (50 ng/ml; R&D Systems), EGF (50 ng/ml, R&D Systems), and IGF1 (50 ng/ml; R&D Systems). Then, 10 μl Cell Counting Kit-8 (CCK-8 Beyotime) per well was added to the 96-well plate. After two hours in the cell incubator, cell numbers were measured using a spectrophotometer at 450 nm absorbance at 0, 12, 24, 36, 48 and 72 hours of culture. Wells without cells served as negative controls.

To examine the chemotaxis and migration activity of CD31^-^CD146^-^ SP cells, 5 × 10^4^ cells were seeded on a Boyden chamber (BD Biosciences) with 8 μm polycarbonate membranes inserted into a 24-well assembly containing EBM2 supplemented with VEGF at a final concentration of 0, 5, 10 or 100 ng/ml, estrogen (Sigma-Aldrich St. Louis, MO, USA) at a final concentration of 0.01, 0.1, 1 or 10 μmol/L and progesterone (Sigma-Aldrich) at a final concentration of 0.03, 0.3, 3 or 30 μmol/L. After 24 hours, the chambers were stained with 0.1% crystal violet and analyzed by photography of the stained cells that had migrated or chemotaxis cells in the lower chamber. The migration or chemotaxis cells were counted in five random fields of vision.

### Endothelial differentiation in vitro

The freshly sorted CD31^-^CD146^-^ SP cells were cultured with EBM2 supplemented with 10% FBS, bFGF (10 ng/ml), and VEGF (50 ng/ml). After 21 days culture through four to five passages, the cells were incubated with mouse anti-human CD31 and mouse anti-human CD146 for 20 minutes at 4°C, then were analyzed on the flow cytometer. To detect the endothelial function, these cells at a seeding density of 2.0 × 105 were seeded on the Matrigel matrix (11.0 mg/ml) (BD Biosciences) in EGM2.

### Transplantation into mouse ischemic hind limbs

The potential of neovascularization of human decidua CD31^-^CD146^-^ SP cells was examined in a murine model of hind limb ischemia in five- to six-week-old nude mice [20]. After subcutaneous anesthesia with pentobarbital sodium, the proximal portion of the femoral artery, including the superficial and the deep branches and the distal portion of the saphenous artery, were ligated. After 24 hours, 100 μl of PBS with or without 1 × 10^6^ freshly detached CD31^-^CD146^-^ SP cells at the third to fifth passage with DiI (Sigma; 10 μM dissolved in dimethyl sulfoxide (DMSO) incubated for 20 minutes) labeling was injected intramuscularly into the distal limb. Then the skin color and temperature, necrosis or growth after ligation of the hind limb was investigated. The mice were fed as before the procedure.

After 21 days, the mice were executed and the muscle tissues of the ischemic hind limb were isolated, fixed and embedded. Serial paraffin sections were stained with mouse anti-human CD54 antibody (Santa) using immunohistochemistry (IHC). The concentration of CD54 was 1:100. The IHC kit was purchased from Zhongshan Biotech Co., Ltd (Beijing, China). Human colorectal carcinoma tissue was used as the positive control and normal mouse immunoglobulin G (IgG) was used instead of monoclonal antibody in the negative control group. The red fluorescent signal of the transplanted cells observed by fluorescence microscopy (Olympus) confirmed the localization of the transplanted cells and their relation to newly formed blood vessels.

### Statistical analyses

Data are reported as means ± SD. *P* values were calculated using the unpaired Student’s *t* test. The number of replicates in each experiment is indicated in the figure legends.

## Results and discussion

### Isolation of CD31^-^CD146^-^SP cells from human first-trimester deciduas

**Figure 1:**
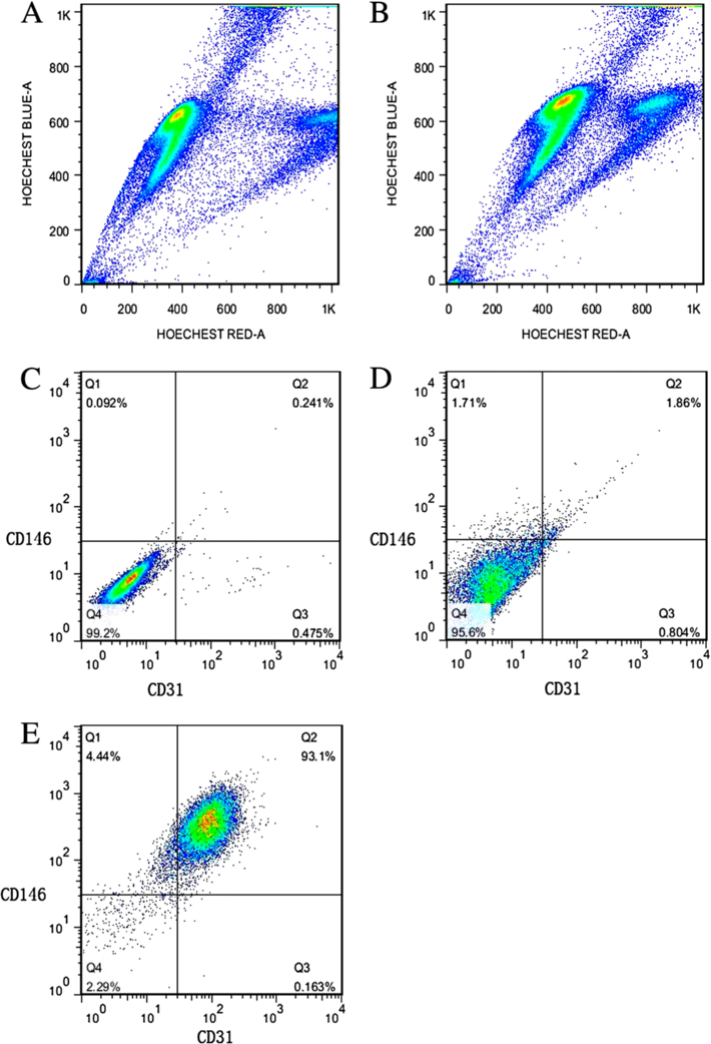
**Flow cytometric analyses for sorting side population cells and its CD31 and CD146 subfraction.** The SP fraction comprised 1.59% of the total cells (**A**), and Hochest 33342 and verapamil staining both ruled out false positive SP cells in the total cells (**B**). This experiment was repeated eight times, and the SP fractions were 1.368 ± 0.393%. The negative control for the CD31CD146 cells (**C**). The CD31^-^CD146^-^ cells comprised 95.6% of the total SP cells and the other three subgroups shared less than 5% of the total SP cells (**D**). The flow cytometric analyses of the cells after being cultured in the differentiation media showed that the CD31^+^CD146^+^ cells comprised 93.1% of the total SP cells (**E**). Data are expressed as means ± SD. The experiments were repeated three times and one representative experiment is presented. SP, side population.

Flow cytometric analyses showed 1.368 ± 0.393% SP granules isolated from human first-trimester decidual tissues (n = 8) (Figures 1A, 1B). By using antibodies against CD31 and CD146, further distinct subpopulations were isolated. CD31 is known to be highly expressed in endothelial progenitor cells and endothelial cells, and CD146 in smooth muscle cells and endothelial cells. The CD31^-^CD146^-^ cells represented 94.51 ± 2.41% of the total SP cells. The other three subgroups were seen in less than 5% of the total SP cells (Figure 1D).

### Proliferation activity, migration of CD31^-^CD146 ^-^SP cells

**Figure 2:**
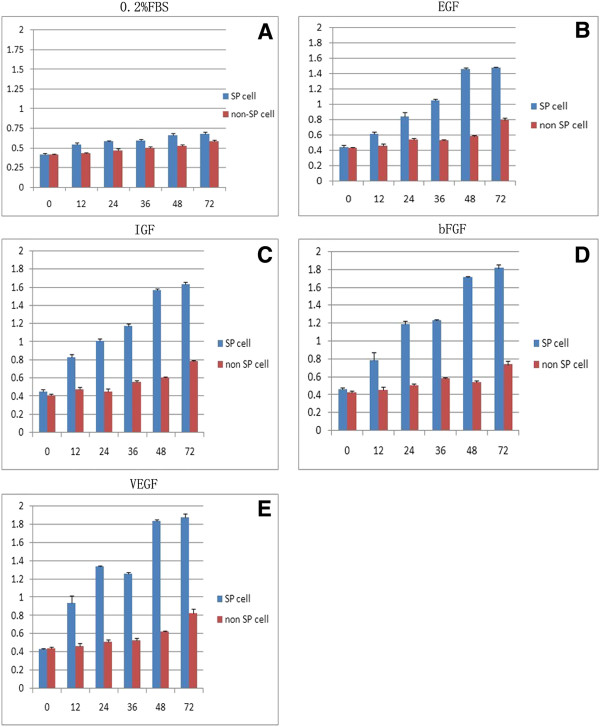
**The proliferation of CD31**^-^**CD146**^-^**SP cells and non**-**SP cells.** CD31^-^CD146^-^SP cells and non-SP cells were treated with 0.2% FBS, VEGF, bFGF, EGF and IGF. All factors can promote CD31^-^CD146^-^SP cell proliferation and have a significant difference between two types of cells (**A**-**E**) (*P* <0.01). Data are expressed as means ± SD. Statistical analysis was performed by the non-paired Student’s *t* test. The experiments were repeated three times and from three patients’ decidua, but one representative experiment is presented. bFGF, basic fibroblast growth factor; EGF, epidermal growth factor; FBS, fetal bovine serum; IGF, insulin-like growth factor G; SP, side population; VEGF, vascular endothelial growth factor.

**Figure 3:**
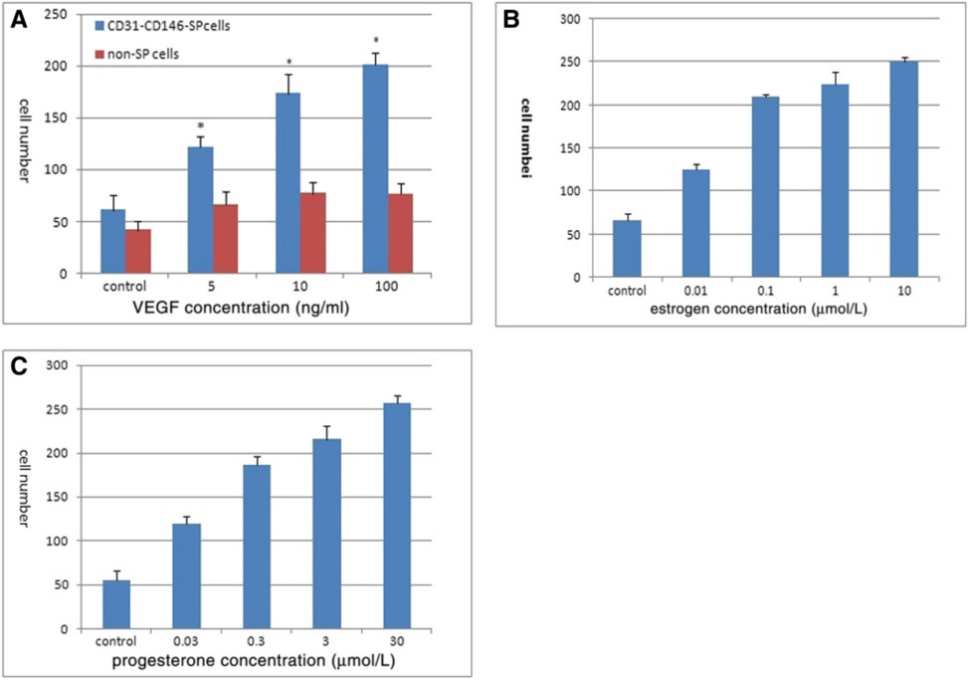
**The migration activity of CD31**^**-**^**CD146**^**- **^**SP cells.** The migration activity with VEGF-A at a final concentration of 0, 5, 10, and 100 ng/ml. Data are expressed as means ± SD (*, *P* <0.01) (**A**). The migration of CD31^-^CD146^-^SP induced by estrogen (10 μmol/L) (**B**) and progesterone (30 μmol/L) (**C**). The minimum concentration of estrogen and progesterone can induce significant migration (*P* < 0.01). Statistical analysis was performed by the non-paired Student’s *t* test. The experiments were repeated three times and from three patients’ decidua, but one representative experiment is presented. SP, side population; VEGF-A, vascular endothelial growth factor - A.

In the presence of different growth factors and sex hormones CD31^-^CD146^-^SP cells and non-SP cell populations proliferated differently. There was a progressive increase with time in the response to the various factors. On day three, VEGF, bFGF, EGF, and IGF treatment enhanced proliferation of CD31^-^CD146^-^SP cells, almost two times more than in non-SP cells and the control treated with 0.2% FBS only (Figures 2A-2E). VEGF was more effective in enhancing proliferation of CD31^-^CD146^-^SP cells compared with the other growth factors at different induction times and inducted CD31^-^CD146^-^SP cell proliferation in a dose-dependent manner (Figure 3A). Estrogen and progesterone at final concentrations of 10 μmol/L and 30 μmol/L, respectively, also promoted the migration of CD31^-^CD146^-^SP cells in a dose-dependent manner (Figures 3B, 3C).

### Differentiation of CD31^-^CD146^-^ SP cells into endothelial cells

CD31^-^CD146^-^SP cells were cultured in the EBM2 supplemented with 10% FBS, 50 ng/ml VEGF and 10 ng/ml bFGF; after 21 days *in vitro* culture, 92.37 ± 2.09% of CD31^-^CD146^-^SP cells expressed both CD31 and CD146 in flow cytometric analyses (Figure 1E).

**Figure 4:**
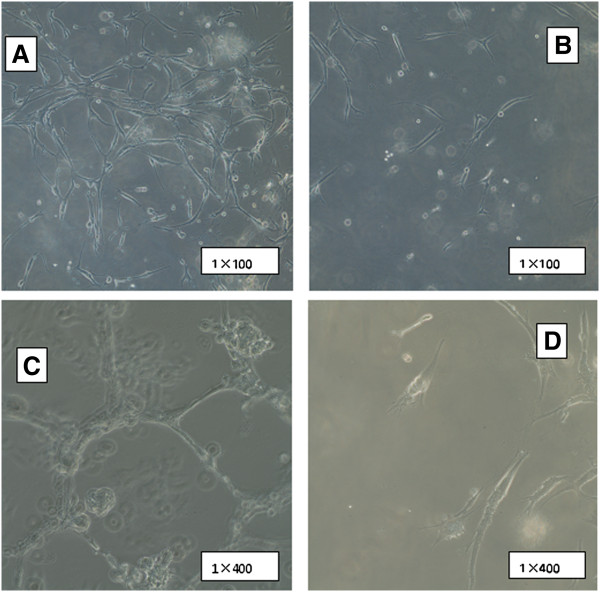
**The tube formation assay for induced CD31**^**-**^**CD146**^**-**^**SP cells 21 days after induction at passage 3 to 4 and non SP cells.** An extensive network of cords and tube-like structures can be seen on Matrigel by induced CD31^-^CD146^-^SP cells after 48 hours (**A**, **C**), and only short strands formed in non-SP cells after 48 hours (**B**, **D**). The experiment was repeated three times, and one representative experiment is presented. SP, side population.

The endothelial differentiation potential was assessed by tube formation assay. Extensive networks of cords and tube-like structures can be observed in induced CD31^-^CD146^-^SP cells cultured on Matrigel as early as 48 hours. The network of cords typically associated with endothelial cells, suggesting an angioblast phenotype (Figures 4A, 4C). Inversely, non-SP cells formed only short strands on Matrigel (Figures 4B, 4D).

### Neovascularization in the ischemic hind limb of nude mouse

**Figure 5:**
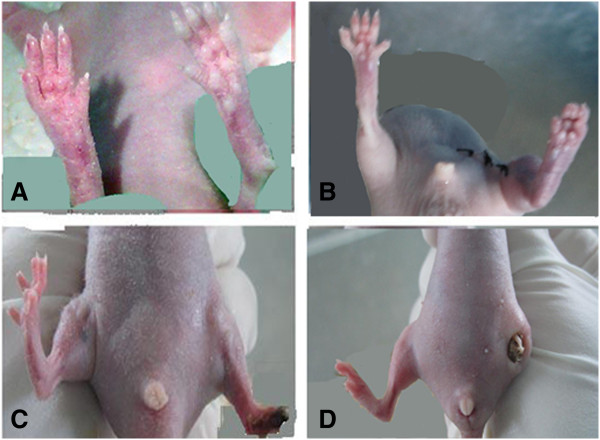
**Nude mouse model (n = 8) of ischemic hind limb and transplanted SP cells (implant group n = 4) or PBS (control group n = 4) after 21 days.** The blood supply of the hind limbs changed immediately after ligation (right) compared with non-ligation (left) (**A**). After 24 hours, the limbs treated by ligation (left) presented necrosis of the extreme limb when compared to the non-ligated limb (right) (**B**). The limbs of the SP cell implant group and the control group are compared (**C** and **D**). PBS, phosphate-buffered saline; SP, side population.

**Figure 6:**
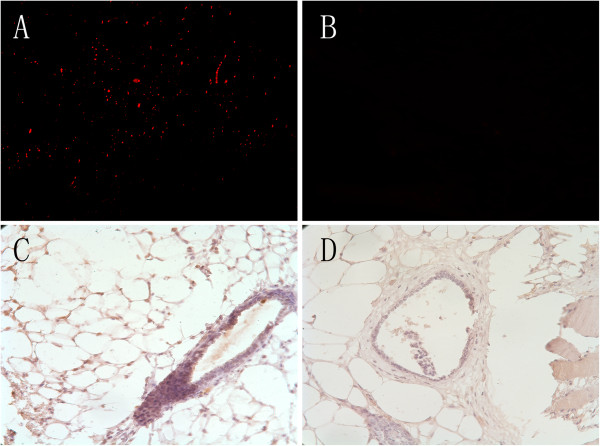
**Dil tracing cells observed under fluorescence microscopy and immunohistochemistry.** The DiI tracing SP cells emerged near vessels and significantly formed part of one vessel in an amputated nude mouse limb (**A**) but there was no signal in the PBS-treated limb (**B**). The positive mouse anti-human CD54 can be found in corresponding DiI positive cells, which indicates that the vessel was composed of differentiated SP cells (**C**); the control limb had no positive signal or staining (**D**). PBS, phosphate-buffered saline; SP, side population.

Immediately after the ligated limbs were cut, the toes of the ligated limbs were pale (Figure 5A). After 24 hours, the limbs treated by ligation exhibited necrosis of limb extremity when compared to the non-ligated limb (Figure 5B). Twenty-one days after transplantation, compared to the limb treated with PBS only (Figure 5D), the left limb of the mice transplanted with CD31^-^CD146^-^SP cells was longer (Figure 5C). We also found cells staining red (tracing the CD31^-^CD146^-^SP cells) in the ischemic hind limb using fluorescence microscopy. These cells were CD 54 positive and formed part of the blood vessel in serial paraffin sections (Figure 6A, 6C) but they did not appear in the PBS-treated limbs (Figures 6B, 6D). Among newly formed capillaries, the number of two positive cells increased demonstrating numerous migrating cells to form new vessels.

The isolatation of the SP cells from hematopoietic stem cells for the efficiency of efflux of the dye Hoechst 33342 accounts for the uniquely low fluorescence of the SP population [13]. This method has been used for the identification of putative somatic stem cells in several human tissues, including human endometrium [15,19,21]–[23]. There is increasing evidence of multilineage differentiation of tissue SP cells including human endometrial SP cells [15,16,24,25]. Cervello *et al*. [26] concluded that the human endometrial SP cells exhibit genotypic, phenotypic and functional features of somatic stem cells, and demonstrated the functional capability of endometrial SP to develop human endometrium after subcutaneous injection in NOD-SCID mice. Furthermore, they found that bone marrow derived stem cells may be a limited source of transdifferentiated endometrial cells, but SP cells in the endometrium maybe play a key role for the mature endometrium [27].

Our study focused on the subfractionation of CD31^-^CD146^-^SP cells from human decidua to assess their differentiation into endothelial cells. Our previous work has proved that SP cells from human decidua can proliferate and form colonies during long-time culture *in vitro* and differentiate into mesenchymal cells [17,18].

It is well known that hemaginoblasts derive from the lateral plate mesodermal and differentiate into endothelial progenitor cells (EPC) and angioblasts derive from intermediate mesodermal and finally differentiate into mature endothelial cells. Usually EPCs and the early angioblasts express CD34, CD133 and VEGFR2, while our data showed that the SP cells did not express the haemopoietic stem cell’s phenotype CD34, CD45, CD133, and CD117, but they expressed CD44, just like the mesenchymal stem cell (MSC) [18]. It has been proven that the MSCs can differentiate into endothelial cells in the uterus [22,28]. So, the SP cells may be the precursors of EPCs. CD31 is expressed during maturation of bone marrow angioblasts to early EPCs and mature endothelial cells [29], and the expression of CD146 increases in the differentiated endothelial cells [30]. In the present study, the induced cells were oblong-shaped and expressed CD31 and CD146 after 21 days. The functional ability of neovascularization of CD31^-^CD146^-^SP cells was determined and it was demonstrated that they formed extensive networks of cords and tube-like structures on Matrigel. Unil now, the decidua vasculogenesis and angiogenesis did not have proper animal models, so we tested the neovascularization function of CD31^-^CD146^-^ SP cells in the mouse hind limb ischemia model. The results implied that they can differentiate to functional endothelial cells and these cells can form vessels *in vivo*. As the remodeling of spiral arteries is important in decidualization, our results show that CD31^-^CD146^-^ SP cells may play an important role in this process and affect the pregnancy outcome.

Recently, Masuda *et al*. [31] have reported that endometrial SP cells have endothelial cell-like properties and can be induced into mature endothelial cells *in vivo* and *in vitro*. Maybe the endometrial and decidual SP cells are the same cells just under a different phase for the endometrium. The differences between these two cells may need more research.

Many studies [32]–[34] have investigated and found the promoting effects of estrogen and progestin on the proliferation, migration and functions of human umbilical vein endothelial cells, EPCs and endometrial endothelial cells. We found estrogen and progestin both promoted the decidua SP cells to proliferate and migrate. Although this effect was in a dose-dependent manner, there was a maximum dose. The increasing of estrogen and progestin along with gestation maybe promote the decidua SP cells to differentiate, proliferate, migrate and finally form functional microvessels in the first trimester.

## Conclusions

As a conclusion, the decidua-derived CD31^-^CD146^-^subfraction of SP cells is vasculogenic, and may induce vasculogenesis *in vivo* in the amputated nude mouse model. Until now, with an understanding of the mechanisms of angiogenesis and the roles of angiogenic factors during implantation, new insights and possible approaches will be provided for embryo implantation and healthy pregnancy.

## Abbreviations

bFGF: Basic fibroblast growth factor; DMSO: Dimethyl sulfoxide; EGF: Epidermal growth factor; EPC: Endothelial progenitor cell; FBS: Fetal bovine serum; FITC: Fluorescin isothiocyanate; IGF: Insulin-like growth factor; IgG: Immunoglobulin G; IHC: Immunohistochemistry; MSC: Mesenchymal stem cell; PBS: Phosphate-buffered saline; PE: phycoerythrin; SP: side population; VEGF: Vascular endothelial growth factor.

## Competing interests

The authors declare they have no competing interests.

## Authors’ contributions

QSW carried out most of the study and drafted the manuscript. LCS participated in the design of the study and performed the statistical analysis. WH participated in the study design and helped to draft the manuscript. YS carried out the immunoassays and cell culture. LX participated in the animal studies and data discussion. WMX participated in the study design and helped to draft the manuscript. LY helped perform experiments. All authors read and approved the final manuscript.
